# The Wyss institute: A new model for medical technology innovation and translation across the academic‐industrial interface

**DOI:** 10.1002/btm2.10076

**Published:** 2017-09-05

**Authors:** Mary Tolikas, Ayis Antoniou, Donald E. Ingber

**Affiliations:** ^1^ Wyss Institute for Biologically Inspired Engineering, Harvard University, 3 Blackfan Circle CLSB5, Boston, MA 02115; ^2^ Vascular Biology Program and Dept. of Surgery Harvard Medical School and Boston Children's Hospital, 300 Longwood Avenue, Boston, MA 02115; ^3^ Harvard John A. Paulson School of Engineering and Applied Sciences, 29 Oxford Street, Cambridge, MA 02138

**Keywords:** biologically inspired engineering, commercialization, nanotechnology, regenerative medicine, synthetic biology, translation, translational medicine

## Abstract

The Wyss Institute for Biologically Inspired Engineering at Harvard University was formed based on the recognition that breakthrough discoveries cannot change the world if they never leave the laboratory. The Institute's mission is to discover the biological principles that Nature uses to build living things, and to harness these insights to create biologically inspired engineering innovations to advance human health and create a more sustainable world. Since its launch in 2009, the Institute has developed a new model for innovation, collaboration, and technology translation within academia, breaking “silos” to enable collaborations that cross institutional and disciplinary barriers. Institute faculty and staff engage in high‐risk research that leads to transformative breakthroughs. The biological principles uncovered are harnessed to develop new engineering solutions for medicine and healthcare, as well as nonmedical areas, such as energy, architecture, robotics, and manufacturing. These technologies are translated into commercial products and therapies through collaborations with clinical investigators, corporate alliances, and the formation of new start‐ups that are driven by a unique internal business development team including entrepreneurs‐in‐residence with domain‐specific expertise. Here, we describe this novel organizational model that the Institute has developed to change the paradigm of how fundamental discovery, medical technology innovation, and commercial translation are carried out at the academic‐industrial interface.

## 1 INTRODUCTION

Engineering has transformed medicine over the past 50 years by applying engineering principles to solve medical problems. This has led to major medical breakthroughs, including heart bypass and dialysis machines, respiratory ventilators, pacemakers, endoscopes, stents, orthopedic implants, drug delivery systems, and various forms of functional imaging, to name just a few. However, engineering, biology, medicine, and the physical sciences are now converging in unexpected ways. Developments in nanotechnology, genetics, synthetic biology, and cell engineering currently allow us to manipulate individual atoms, genes, molecules, and cells one‐at‐a‐time, and even create artificial living cells. At the same time, progress in materials science, computer science, stem cell biology, and tissue engineering has enabled scientists to create adaptive self‐assembling materials, microdevices, and biomolecular computational strategies to manipulate cell function, control complex organ physiology, guide tissue regeneration, and engineer artificial tissues and organs for applications ranging from regenerative medicine to diagnostics and therapeutic discovery. The reality is that engineers and physicists now commonly publish in biological journals, while biologists and physicians submit articles to engineering and physics publications, and computer scientists integrate their work into all forms of biomedical research. As a result, the boundaries between living and nonliving systems are literally breaking down.

The Wyss Institute for Biologically Inspired Engineering at Harvard University^1^ was founded in 2009 based on the belief that we have uncovered enough information about how Nature builds, controls, and manufactures from the nanoscale to the macroscale that we can now leverage these biological principles to develop new engineering innovations. We named this burgeoning field, “Biologically Inspired Engineering,” to clarify how it differs from the more established disciplines of Bioengineering and Biomedical Engineering that continue to apply engineering principles to address biomedical challenges.

The Wyss Institute's mission is to discover the biological principles that Nature uses to build living things, and to harness these insights to create biologically inspired engineering innovations to advance human health and create a more sustainable world. Its foundation lies in the recognition that breakthrough discoveries cannot change the world if they never leave the laboratory. And thus, the Institute was designed to confront the challenge of translating technology advances across the academic‐industrial interface, as much as it was created to catalyze the emergence of Biologically Inspired Engineering.

The Institute is similar to other academic research institutes in that it resides within the academic ecosystem of Harvard University. But the structure of the organization is specifically designed to overcome many of the challenges that in the past have held back technology innovation, intellectual property generation, and the commercialization of academic discoveries. This is crucial because these translation challenges continue to be a major stumbling block for universities and hospitals as they attempt to transition the discoveries their faculty and staff make into the marketplace where they can improve patients' lives. In this article, we provide an overview of the organizational model we have developed to enable cross‐disciplinary research collaborations, support technology innovation, augment intellectual property generation, and ensure efficient translation of discoveries into products that enter the marketplace in order to enhance human health.

## 2 A NEW PATH TO ACADEMIC INNOVATION WITH A TRANSLATION MISSION

Companies tend to be optimized to generate intellectual property, protect its value, and develop products efficiently; however, as they are primarily focused on maximizing shareholder value, they often do not support pursuit of high‐risk ideas that arise in their organizations. Difficulties in fostering innovative technology development are typically compounded in industry by most companies' unwillingness to change market direction and refocus operations due to the large capital investments they often have made for scale up and manufacturing of existing products in already well‐defined market segments. In contrast, academia is a cauldron of innovation and creativity, but the focus is on publications with little focus or investment in intellectual property creation and its translation into useful products; thus, only few research advances have any direct impact on society at large.

The Wyss Institute was launched with a $125 million gift to Harvard University from Hansjörg Wyss, which at the time was the largest single philanthropic gift in the University's history, and this was supplemented by contribution from Harvard as well. Based on our success within 5 years, our donor doubled his gift to $250 million. As a philanthropic entrepreneur who had been the CEO of a large medical device company, the donor shared this view and noted that “Big companies will not take risks. I try to convince my people to change directions, and they do not respond; it's like trying to turn a huge tanker. You academics do innovative things, but all you do is publish papers and make widgets. What I would like to see is an innovative startup in the world's greatest academic environment that will take risks and have positive near term impact on the world.”

As a result, the Wyss Institute has pioneered a new model that combines the focus on applications, intellectual property generation, and commercialization commonly found in a start‐up with the creative freedom and flexibility of academia. The measures of success that were established when the Institute was formed included academic ones, such as international recognition for our scientific leadership, and recruitment of world‐class faculty, students, and fellows. However, we are also assessed based on the patent portfolio we generate and our ability to develop productive corporate alliances, licensing agreements, and new start‐ups. Having our bioinspired technologies being sold or productized to achieve near‐ and long‐term impact is the ultimate measure of the Institute's success.

In traditional academic settings, graduate students, postdoctoral fellows, and young faculty are not commonly trained to patent the technologies they invent, and their career advancement is not linked to their success at reducing innovations to practice. This is one of the major reasons why academic research produces a huge amount of information and innovations that are potentially exciting, but they rarely leave the laboratory, resulting in little or no positive impact on our broader society. The Wyss Institute aimed from its beginning to address this gap.

Given this focus, one of our major goals was to create a rich environment that is specifically designed to support interdisciplinary research and intellectual property generation, as well as prototype development, testing and validation, thereby de‐risking technologies both technically and commercially. Our belief was that in addition to generating stronger intellectual property, successful maturation and de‐risking of technologies led by a multidisciplinary collaborative team of world‐leading scientists and engineers would attract funding from both industry and government agencies that are focused on development of disruptive technologies (e.g., DARPA, IARPA, DoD, etc.), thereby increasing the likelihood that these innovations will transition to the marketplace. However, to accomplish this, we realized that we must build our community from the bottom‐up, because all creative endeavors are based on people.

To achieve these unique goals within an academic setting, the Wyss Institute was set up to span all Harvard schools and departments, and not be confined within any one school. In fact, it became a nonprofit 501(c)3 corporation inside Harvard University with its own governance structure in 2013. The Institute's Board of Directors, which includes both Harvard and external members, is tasked with the overall governance of the Wyss Institute. Programmatic decisions are made by the Institute's Operating Committee, comprised of the faculty leads from each of the six enabling technology platforms described below, ensuring that faculty members are in control of the programmatic direction of the Institute. Through this governance structure, the Institute has been able to establish semiautonomous research and administrative operations, policies, and procedures that are both consistent with those of Harvard, while meeting the Institute's needs for flexibility and adaptability. Institute administrative staff are recruited with a strong emphasis on the fit with the Institute's entrepreneurial, collaborative, and service‐oriented culture, which results in high‐performing administrative teams similar to those more commonly seen in start‐ups, which focus on advancing the Institute's mission by specifically meeting the needs of the Institute's faculty and researchers. Institute staff crisscross the university and partner institutions, and work collaboratively with staff at various Harvard schools to administer interschool academic appointments, manage research, ensure compliance, and oversee sponsored funds and finances. The Institute is not an academic appointing unit, so all of its faculty have academic appointments in their home institutions, and most faculty (except for new junior hires) maintain research laboratories at their home institution, while also conducting research at the Institute site. This is a subtle but important part of our recipe for success: faculty can create their own “empires” that reflect their own unique approaches in their home laboratories, but they learn that they need to pursue a different and highly collaborative approach, consistent with our more entrepreneurial culture, if they want to establish operations at the Institute site and become an active member of our community.

The Institute currently has more than 350 full‐time staff members, including 20 core and 16 associate faculty members, and close to 40 scientists and engineers recruited from industry, who bring significant experience in product development from a broad range of medical and nonmedical markets into our community. Core faculty members are appointed for 3‐year renewable terms, while associate faculty has 1‐year renewable appointments. Fellows and students from different faculty laboratories are brought together and co‐located at the main Institute's site in the center of Boston's Longwood Medical Area, and at a satellite site on Harvard's Cambridge engineering campus. In addition, we harness the deep academic resources and talent, clinical depth, and broad technology expertise of the entire Greater Boston/Cambridge region by having established a consortium of collaborating institutions, including Harvard University and its affiliated hospitals (Beth Israel Deaconess Medical Center, Brigham and Women's Hospital, Boston Children's Hospital, Dana Farber Cancer Institute, Massachusetts General Hospital, and Spaulding Rehabilitation Hospital), as well as Boston University, Massachusetts Institute of Technology, Tufts University, and the University of Massachusetts Medical School. In the last few years, we expanded the roster to include the University of Zurich and Charité Berlin based on collaborations with faculty from these institutions that emerged from common interests and bottom‐up involvement with our core faculty.

The consortium is structured so that faculty and staff from all these institutions and diverse disciplines can work side by side at the Institute's sites. Although we primarily focus on medical challenges, we realized that there are many technologies that exist in nonmedical areas, (3D printing‐based manufacturing, for example), which could add huge value for medicine, and there are biomedical technologies (e.g., synthetic biology) and approaches that could be equally valuable for nonmedical fields ranging from energy and manufacturing to data storage. Thus, our Institute's research areas include nonmedical pursuits focused on environmental sustainability, as well as more classical biomedical research challenges. Also adding to the richness and reach of the Institute are its strong collaborations with industrial partners who offer the understanding of markets and user needs to match the breadth of applications envisioned by Institute researchers. This constant flow of people between the Institute and its many collaborating partners helps to maintain a multifaceted exchange of information, ideas, and resources, while consolidating our focus on biologically inspired engineering across the Greater Boston region and beyond.

## 3 MULTIDISCIPLINARY RESEARCH AT A HIGHER LEVEL

We launched the Institute by enlisting 14 of the most brilliant, innovative, and entrepreneurial faculty from Harvard University and our collaborating institutions as our founding core faculty members and over time augmented this group with new recruitments of both core and associate faculty (Table 1).^2^, ^3^ The breadth of interests and expertise of these faculty are enormous, ranging from synthetic biology, cell biology, physiology, and many areas of clinical medicine and surgery to inorganic chemistry, self‐assembly, polymer physics, nanotechnology, microfabrication, robotics and computer science, as well as virtually all fields (mechanical, chemical, biological, electrical, cell, tissue, organ) of engineering. However, what truly raised our cross‐disciplinary effort to a higher level was the recruitment from industry of close to 40 technical staff scientists and engineers, or members of what we call our “Advanced Technology Team” (ATT),^4^ who each bring years and even decades of prior industrial experience in product development into our community. These ATT staff members do not run core facilities as is done at many other major research institutes; instead, they are integrated into our research teams along with faculty, fellows, students, and other technical staff. ATT members have been recruited from a variety of industrial sectors and companies ranging from major pharmaceutical and biotechnology companies, such as *Pfizer, AstraZeneca, GlaxoSmithKline, Vertex,* and *Biogen* to *Procter & Gamble, Dow Chemical, Pratt & Whitney, IBM, Analog Devices, iRobot, Sonos*, and even *Ben Hogan Golf Equipment Company*.

**Table 1:** Wyss Institute Core Faculty since its founding

Wyss core faculty		
Joanna Aizenberg, PhD*	George Church, PhD*	James J. Collins, PhD*
SEAS, FAS	HMS	MIT (originally BU)
David A. Edwards, PhD*	Ary Goldberger, MD*	Donald E. Ingber, MD,PhD*
SEAS	BIDMC, HMS	BCH, HMS, SEAS
**Neel S. Joshi, PhD**	**Jennifer A. Lewis, ScD**	L. Mahadevan, PhD*
SEAS	SEAS	SEAS, FAS
**Samir Mitragotri, PhD**	David J. Mooney, PhD*	Radhika Nagpal, PhD*
SEAS	SEAS	SEAS
Kevin Kit Parker, PhD*	William Shih, PhD*	Pamela Silver, PhD*
SEAS	DFCI, HMS	**HMS**
**David R. Walt, PhD**	**Conor Walsh, PhD**	**David A. Weitz, PhD**
BWH, HMS	SEAS	SEAS, FAS
George M. Whitesides, PhD*	Robert Wood, PhD*	**Peng Yin, PhD**
FAS	SEAS	HMS

Asterisks indicate 14 founding core faculty members. Affiliation abbreviations: BCH, Boston Children's Hospital; BIDMC; Beth Israel Deaconess Medical Center; BWH, Brigham and Women's Hospital; BU, Boston University; DFCI, Dana Farber Cancer Institute; HMS, Harvard Medical School; SEAS, Harvard John A. Paulson School of Engineering & Applied Sciences; FAS, Harvard Faculty of Arts and Sciences; and MIT, Massachusetts Institute of Technology.

The point is that when one of our scientists raises a medical technology challenge, such as the need for a new material with particular physical properties, the answer might readily come from one of our ATT members who has worked in an entirely different field and has encountered a material with similar properties in a completely different application. This capability, combined with the experience of our ATT members in developing timelines and meeting milestones in an efficient way, has enabled Institute researchers to innovate and meet program goals at a pace and focus that are rarely seen in academia. In addition to bringing extensive skills in product development and team management from various industrial sectors, the ATT members help build and lead integrated technology development teams focused on high‐value applications. They also catalyze communications and interactions across the Wyss Institute to ensure that we remain at the leading edge of technology translation.

The Wyss Institute also breaks down the institutional “silos” by organizing its physical space into “Collaboratories.” First, we do not provide faculty members with their own independent research laboratories. Instead, our “Collaboratories” define a cohesive, open physical footprint where fellows, students, and staff from different faculty groups, ATT members and other relevant Institute staff co‐locate to work on a common high value project application or major platform area of the Institute, examples of which are described below. In addition, we built cutting‐edge capabilities that are accessible by all members of our community. One example is our unique machine shop, which is unlike most found in academia (and likely none in a medical school) as it is staffed by experienced, full‐time engineers and houses state‐of‐the‐art manufacturing equipment that enables fast prototyping and construction of virtually any type of device from the microscale to the macroscale. We also can build using a broad range of materials including those that are approved by the FDA for various medical applications. These capabilities allow members of our community to innovate and iterate at a rate that is uncommon for academia, which greatly enhances our ability to meet and beat milestones on both government‐ and industrial‐funded technology development projects.

## 4 THE INSTITUTE'S PLATFORM FOCUS AREAS AND INITIATIVES

The Wyss Institute's research and development efforts are currently organized around six enabling technology platforms and two cross‐platform initiatives,^1^ each composed of teams of institute faculty, students, fellows, and staff who develop the new cutting edge technological capabilities necessary to enable a new wave of bioinspired materials and devices. These eight focus areas provide the Institute and its collaborators with unique technical resources and state‐of‐the‐art equipment, as well as a rich, open, interdisciplinary environment for the students and staff to learn how to translate ideas and discoveries into products with great clinical or commercial value. Interestingly, even the way in which we create these research platforms and initiatives is bio‐inspired in that they are self‐organizing, dynamic, and constantly evolving based on the shifting intellectual interests of our community members as well as the technical needs and challenges of the wider commercial marketplace.

Platform and project self‐assembly at the Institute results from the free flow of ideas and interactions among faculty and researchers, as well as administrative and business development staff. It is amplified by a culture that welcomes exploration and taking risks, which allows our staff and researchers to pursue their “out of the box” ideas without the stigma and fear of potential failure. Through its philanthropic funds, the Institute has the ability to readily support such ideas at their nascent stage, without the need for internal grant applications that typically stifle the excitement in organizations through long and bureaucratic decision points. Some of these ideas arising in our community may “fizzle out” as the first experiments may fail in the lab or the team dynamics may not survive the early stages of formation. But some ideas and teams gain momentum, and with increasing technical success comes the need for additional resources and focus. The Institute will then leverage government and industrial funding, in addition to internal platform funds, to further de‐risk technologies, moving them from concept to prototype in specific applications and fields.

Our current research focus areas include:

- **Adaptive Material Technologies.** The goal is to understand and apply principles of self‐assembly and self‐organization—nature's own principles—to create adaptive, responsive, and self‐optimizing materials, devices, and structures that adapt their shapes and functions to provide properties critical to sustainability. Although the focus is on nonmedical areas, materials developed by pursuing this focus include self‐healing, repellant coatings for the prevention of infectious biofilm formation on implantable medical devices^5^ (Figure 1) and liquid‐gated membranes that can selectively process complex material flows,^7^ precisely separating liquids, gases, and solids without clogging and with significant energy savings, which could have significant value for pharmaceutical manufacturing.
  **Figure 1:**
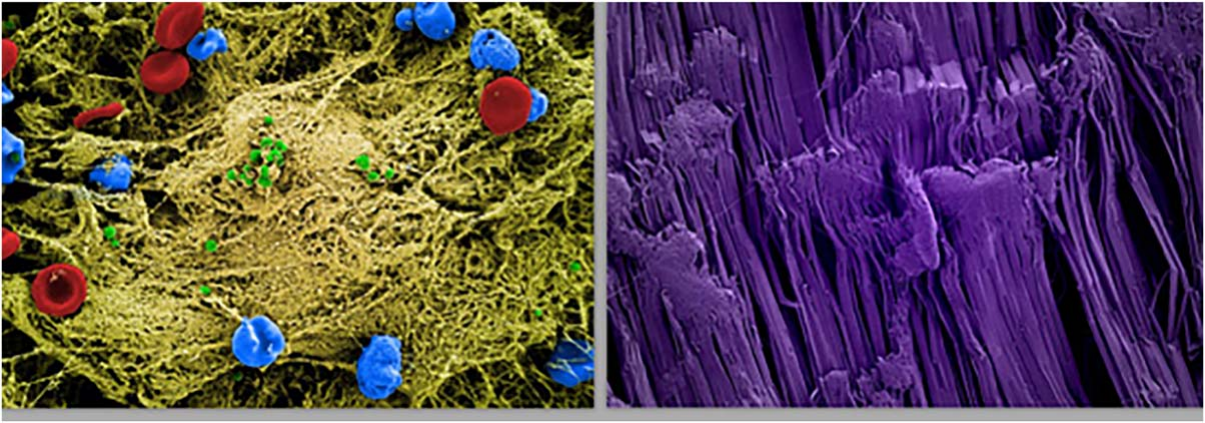
A bioinspired surface coating for medical devices that inhibits bacterial biofilm formation. Scanning electron microscopic images that show the surface of teflon devices that were implanted into mice infected with *S. aureus* (green) without (left) or with (right) a pretreatment with “SLIPS” (Slippery Liquid‐Infused Porous Surface) coating (erythrocytes, red; immune cells, blue; extracellular matrix (yellow). [Work from Joanna Aizenberg and the Wyss Adaptive Materials Platform]^6^
- **Bioinspired Soft Robotics.** The aim is to create mobile, soft, and wearable robotic devices that move, adapt, and work collaboratively or seamlessly integrate with the human body. A major effort has led to the development of soft wearable devices, including lightweight exosuits (Figure 2) that combine classical robotic design and control principles with functional apparel to increase the wearer's strength, balance, and endurance. The exosuits are currently being commercialized as a way to normalize gait in patients with stroke, but can find application in nonmedical areas, such as sports, as well as recreation and consumer markets.^8^
  **Figure 2:**
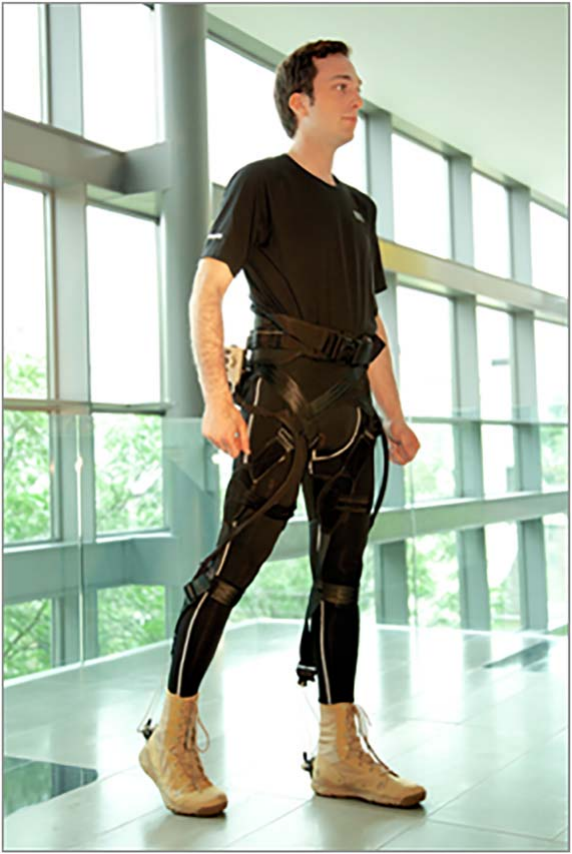
Soft exosuits for gait enhancement. Lightweight and flexible exosuits made of fabrics with integrated artificial muscles and robotic control offer a new way to assist children and adults with movement disorders such as stroke, multiple sclerosis, and Parkinson's disease, the elderly in maintaining or restoring gait or soldiers, firefighters, paramedics, and others whose jobs require them to carry extremely heavy loads. [Work from Conor Walsh and the Wyss Bioinspired Robotics Platform]^8^
- **Biomimetic Microsystems.** This effort is currently focused on therapeutics discovery and drug delivery enabled by microsystems technologies. One major success area has been the development of human Organ‐on‐a‐Chip microfluidic cell culture devices (Figure 3) that recreate the tissue‐tissue interfaces, physical microenvironment, and vascular perfusion of living human organs, including the lung, intestine, kidney, bone marrow, liver, skin, heart, muscle, and brain.^9^ Institute researchers are currently leveraging this novel ability to carry out human experimentation in vitro to develop improved models of human diseases, engineer new targeted delivery technologies, and identify novel therapeutic targets for diseases, such as pulmonary edema, chronic obstructive pulmonary disease, inflammatory bowel disease, malnutrition, and kidney glomerulopathies. Another aspect is leveraging the Institute's pathogen capture platform that uses a genetically engineered version of a natural human blood opsonin protein to develop new diagnostics and therapies to treat infectious disease, blood infections, and sepsis.^11^ Other efforts include discovery of new therapeutics for enhancing host tolerance to infection as well as radiation injuries, and identifying enhanced methods for targeted organ‐specific delivery across biological interfaces, such as the human blood‐brain barrier.
  **Figure 3:**
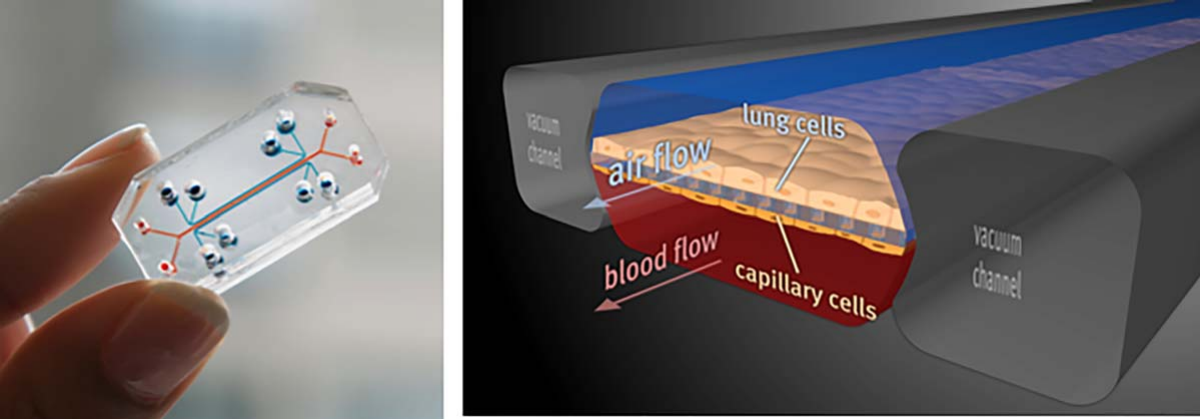
Human organs‐on‐chips. Each individual organ‐on‐chip is a microfluidic cell culture device composed of a clear flexible polymer about the size of a computer memory stick (left) that contains hollow microfluidic channels lined by living human cells interfaced with a human endothelial cell‐lined artificial vasculature (right). Mechanical forces can be applied to mimic the physical microenvironment of living organs, including breathing motions in lung, peristalsis in the intestine and pulsations due to blood flow in the kidney. Organs‐on‐chips, which have been developed as replacements for animal testing, have been shown to recapitulate the physiology of many different organs, mimic complex disease states, and predict drug responses as well as toxicities. These organs are also being linked together by their vascular channels to create a human body‐on‐chips to predict drug pharmacokinetic and pharmacodynamic behaviors in humans. [Work from Donald Ingber and Kevin Kit Parker and the Wyss Biomimetic Microsystems Platform]^9^, ^10^
- **Immuno‐Materials**. Institute researchers are developing material‐based systems capable of modulating immune cells ex vivo, as well as within the human body, to treat or diagnose disease. Examples of immune‐modulating technology development efforts include an implantable, biodegradable cancer vaccine that uses a polymer scaffold containing growth factors and components of a cancer patient's tumor to recruit and reprogram the patient's immune cells “on site” and kill cancer cells (Figure 4), which is currently being evaluated in a Phase I human clinical trial.^12^ An injectable broad‐spectrum infection vaccine is also being developed, as well as implantable biomaterials that concentrate and trap disease‐related circulating T cells, which can be retrieved to gain insights into the mechanisms of action and toxicities of novel therapeutics.^13^
  **Figure 4:**
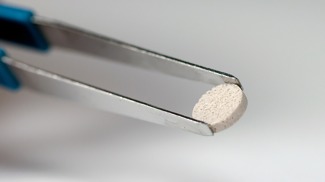
Implantable cancer vaccine. This technology consists of a biodegradable polymer scaffold containing cytokines, immune adjuvants, and antigen components from each patient's tumor. It is implanted subcutaneously at a site distant from the tumor, and works by recruiting dendritic cells and reprogramming the immune system to kill the cancer cells. The cancer vaccine is currently in Phase I human clinical trials and this general approach one day also could help overcome infectious diseases and auto‐immune diseases, as well as vaccinate against specific peptides, proteins, or antigens. [Work from David Mooney and the Wyss Immuno‐Materials Platform]^12^
- **Living Cellular Devices**. The goal here is to re‐engineer living cells and biological circuits to create programmable devices for medicine, manufacturing, and sustainability. An application of such devices is in the diagnostics space where synthetic gene networks composed “circuits” of re‐engineered molecular components have been created to detect the genetic signatures of RNA viruses, such as Ebola and Zika, within one week after the causative agent is identified (Figure 5).^14^ On the therapeutics side, Institute researchers have engineered a genetically mutated version of erythropoietin with a weakened ability to bind to its natural receptor, thereby preventing binding to unwanted cell types and reducing potentially toxic side effects.^15^ Other efforts focus on reengineering of probiotic microbes that can act as sentinels of infection or as therapeutic factories when ingested and grown inside our intestines.
  **Figure 5:**
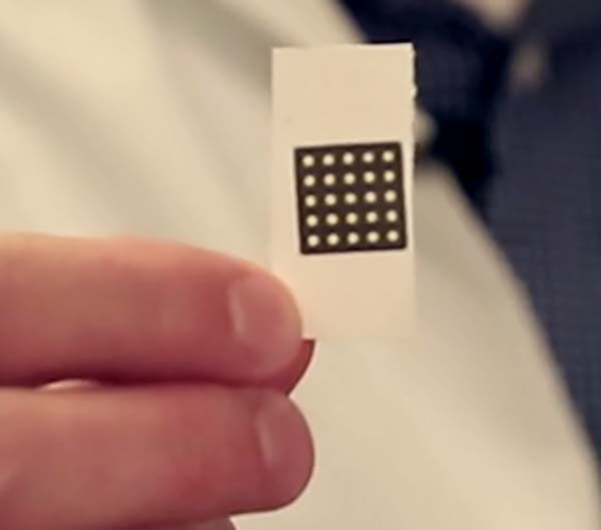
Rapid Zika Diagnostic. Rapid, cheap, accurate diagnostics, and sensors are built using synthetic gene networks and ordinary paper, allowing for storage and safe transport beyond the lab. [Work from James Collins and the Wyss Living Cellular Device Platform]^14^
- **Molecular Robotics.** This initiative leverages recent advances in bioinspired molecular engineering pioneered at the Institute, such as DNA origami (Figure 6) to develop programmable, self‐assembling swarms of molecular scale robots that can be directed to collectively carry out specific tasks without the need for power, wires, or batteries.^17^ This is being accomplished by combining approaches from molecular scale DNA/RNA engineering from synthetic biology with bioinspired distributed algorithms from swarm robotics. The programmable, biocompatible nanobots can be designed to impact a broad range of fields that rely on design and analysis at microscopic scales, including single protein sequencing, highly multiplexed high‐resolution imaging for pathological analysis, and drug delivery.
  **Figure 6:**
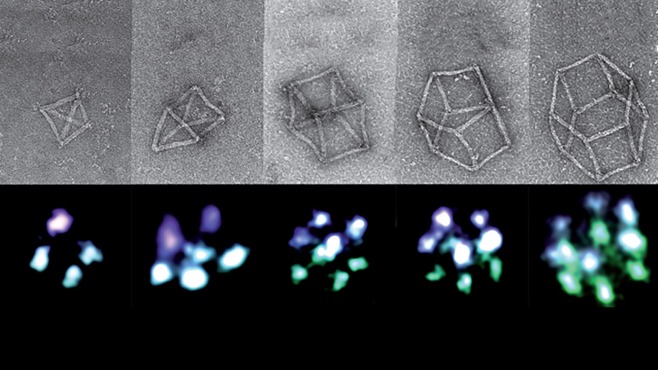
3D structures self‐assembled from programmable DNA. Electron micrographs (top) showing that nanostructures with any desired three‐dimensional shape can be built using DNA that can be programmed to self‐assemble on command, leveraging DNA's known base pair binding strategies. This approach, which enables precisely placement of molecular cargoes such as fluorescent beacons at predetermined locations on the structures (bottom), is being leveraged to develop new approaches to nanofabrication, optical detection, molecular analysis, and drug delivery. [Work from Peng Yin and the Wyss Molecular Robotics Initiative]^16^
- **Synthetic Biology.** The focus here is on developing breakthrough approaches to reading, writing, and editing nucleic acids and proteins for multiple applications, ranging from healthcare to chemical manufacturing. Gene Drives,^18^ CRISPR technology for mammalian cells, and highly multiplexed fluorescent in situ sequencing^19^ (Figure 7) are a few examples of disruptive breakthroughs that have emerged from Institute efforts. By applying novel methods for large‐scale, affordable DNA synthesis, researchers at the Institute are also now using DNA to create a long‐lasting, stable, and extremely dense format for information storage.^20^
  **Figure 7:**
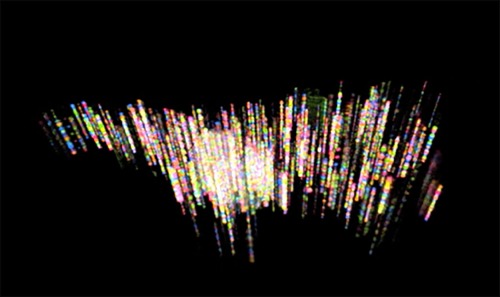
Fluorescence in situ sequencing (FISSEQ). This highly multiplexed imaging‐based method allows scientists to pinpoint the location of thousands of RNAs at once within intact cells and tissues, and simultaneous determine each of their sequences at those locations inside the cell. The method is carried out with enzymes that copy each mRNA into a matching strand of DNA “on‐site,” multiplying that DNA strand many times to create tiny balls of replica DNA, still fixed to the original spot. Using four different fluorescent dyes—one for each of the DNA's four bases—a sequence of flashing colors reveals each replica DNA's exact sequence under a super‐resolution microscope, as shown here in this computer‐reconstructed 3D image of sequences distributed within a single cultured cell. FISSEQ could lead to new diagnostics that spot the earliest signs of disease, and help reveal how neurons in the brain connect and function. [Work of George Church and the Synthetic Biology Platform]^16^
- **3D Organ Engineering**. This is our most recent initiative with a focus on engineering functional, multiscale, vascularized organ replacements that can be seamlessly integrated into the human body. The highly interdisciplinary collaborative teams are leveraging new advances in 3D bioprinting (Figure 8), microfabrication, and the Institute's extensive knowhow relating to construction of human Organs‐on‐Chips to create entirely new approaches to fabricate functional human organ structures for both in vivo and extracorporeal therapeutic applications.^22^
  **Figure 8:**
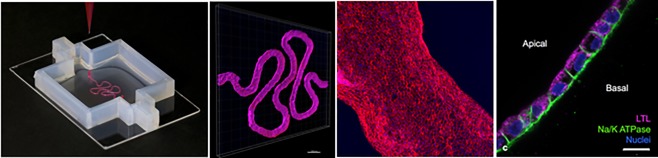
3D bioprinting of living tissues. A 3D Bioprinter has been developed that enables printing of biocompatible hydrogels containing living human cells, which has been used to build a human kidney proximal tubule. (Left) A photograph of the tubule structure being fabricated on the 3D printer with a collagen gel; (Left middle) A 3D confocal microscopic reconstruction of the engineered tubule structure lined by fluorescently labeled human proximal tubular epithelial cells; (Middle right) A high magnification fluorescence microscopic image of the engineered tubule showing that it is lined by a continuous layer of fluorescent epithelial cells; and (Right) A higher magnification fluorescence microscopic cross‐sectional view through the engineered tubule demonstrating that it is lined by a continuous polarized kidney proximal tubular epithelium (e.g., expressing Na+/K+ ATPase only on its basolateral surface). [Work of Jennifer Lewis and the 3D Organ Engineering Initiative]^21^

## 5 THE INSTITUTE'S TECHNOLOGY TRANSLATION MODEL

**Figure 9:**
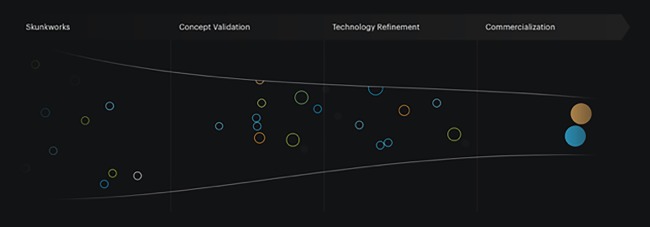
The institute's innovation funnel. The funnel maps the institute's technology development and translational activities from idea conception through commercialization^1^

We map all of our translational activities, from idea conception through early technical development to further technical de‐risking, business development, intellectual property protection, and eventual commercialization, onto a “Technology Innovation Funnel”^1^ (Figure 9). At the broadest part of the funnel, we harness the creative freedom of academia to generate a pipeline of new ideas and potential breakthrough technologies, focus these ideas, and move them towards Concept Refinement and Prototype Optimization in collaboration with end‐users, clinicians, regulatory agencies, and industrial partners who help us to best understand the market needs and problems early in this process and to inform our approach to technology development.

Most researchers do their most creative work in the “spaces between grants” because government funding often supports only incremental advances, and they often generate their most exciting and visionary ideas long after grant applications are submitted. We turbo‐charge this approach by providing salary and supply funding to each of our core faculty members which allows them to hire two students or fellows who can pursue any creative technology concept or translation idea that is relevant to the field of biologically inspired engineering. We also teach our students, fellows, staff and faculty to disclose reports of invention (ROIs) as early in the development process as possible. These ROIs are reviewed by an intellectual property attorney who is employed full‐time and is located onsite at the Institute. The feedback provided by this attorney, combined with input from ATT members who have technology‐relevant industrial expertise, and from business development staff who are hired by the Institute or work within Harvard University's Office of Technology Development (OTD), help to focus the investigator's research efforts so that they pursue the shortest path towards developing new technologies that have the greatest chances for commercial success, and hence, the highest likelihood of reaching patients.

Business and intellectual property portfolio development at the Institute are as critical to the Institute's mission of translation as is the development of new technologies. Because all intellectual property generated at the Institute sites is owned and managed by Harvard University, decisions relating to intellectual property and business development are made through a collaboration between Institute business development staff and members of Harvard's OTD, some of whom are also funded by the Institute to ensure their dedicated focus on Institute‐related technologies. All Institute‐funded staff who manage technology translation activities are located at the Institute site to ensure their effective integration with the faculty, research teams and technology development efforts at the Institute.^23^

Technologies that pass successfully through technology prototyping and validation, and progress towards the narrow end of the funnel, are sometimes licensed to established companies or new startups, much like in any other academic institution. However, some ideas emerge from this technology pipeline and progressively mature into higher value programs that garner even greater external investor interest because of positive end‐user feedback supporting the high potential value and impact of the technology. With this type of high value technology, we often receive input from investors that they want to see the technology de‐risked further, both technically and commercially, before they will invest. In cases where we have this type of established interest from investors and end‐users, a high performing and passionate Institute team championing the technology (often with members who have prior industrial experience), and strong intellectual property and technical data, the Institute establishes what we call “Institute Projects.” These programs provide the additional financial support and access to facilities or knowhow required to de‐risk the technology, cross the “valley of death,” and launch a new commercial entity with a higher likelihood of success than is typical in academia. Furthermore, unlike the conventional academic model of translation, where licensing patents directly from the academic lab into a commercial entity has given rise to the “valley of death,” the Institute's approach offers a key advantage in its ability and willingness to file IP and hold on to it until its value is better established. This more strategic approach increases both the new venture's probability of success and the initial valuation of the technology.

By bringing in entrepreneurs‐in‐residence (EIRs) with relevant, application and industry‐specific experience, and teaming them with the faculty inventors and technical champions of each technology, we have been able to nurture these Institute Project efforts as “virtual startups” within the Wyss Institute environment. For example, under the guidance of EIRs, our funding has been used to support large animal efficacy and toxicity studies for specific clinical indications; conduct market research and interview multiple physicians and government officials around the world to identify challenges that must be overcome for the development, deployment, and reimbursement of a new infection diagnostic; reduce the manufacturing costs for a vibrating insole that provides elderly with the balance of a 20‐year old and carry out a clinical validation study; and even initiate a Phase I human clinical trial for a cancer vaccine in collaboration with our consortium member, the Dana Farber Cancer Institute in Boston. The concept of a focused Institute Project with well‐identified goals and milestones allows us to significantly mature and de‐risk our most valuable and disruptive technologies, while developing a putative leadership team, which can increase the startup valuation when these projects spin out. This approach also allows them to hit the ground running with an experienced team already in place, thus greatly increasing their likelihood for long‐term commercial success. Institute Projects that do not lead to a new startup are either eventually licensed, or they come to an end at the Institute.

The creation and implementation of our model of translation inside Harvard's well‐established academic environment has had its challenges. The Institute had to find ways to develop its own unique approach to translation and creation of an entrepreneurial culture, while maintaining close connections to the rest of the university and respecting its policies. Such co‐existence requires a clear definition of the Institute's goals and culture, consistently articulated and communicated to all stakeholders from faculty to staff to collaborators alike, as well as clarity on setting internal priorities and making choices consistent with these goals and culture. Maintaining this focus in the presence of traditional academic pressures has been and continues to be a challenge. We also have faced other challenges related to effectively balancing the open innovation and academic “skunkworks” of academia with the market focus and disciplined development required to create and de‐risk technologies that can eventually become viable entities of their own. Moreover, the Institute is a dynamic entity, continuously managing the flow of people and ideas as technologies mature and move out. However, establishment of this “dynamic equilibrium” has a major advantage as well, as it enables us to make room for new ideas and people to take their place when startups fold out of the Institute, thus allowing us to continually change focus and keep the Institute vibrant. This process of continuous regeneration presents a challenge that requires a management approach that is flexible yet truly engaged with all aspects of the organization.

## 6 INSTITUTE IMPACT

During its brief eight and one‐half year history, the Institute has achieved a number of important milestones and successes and has consistently met all of its measures of success, both academic and commercial. Our faculty have received numerous awards, including many being elected as members of the National Academies of Science, Medicine, or Engineering. While most of our faculty members only maintain portions of their research staff at the Institute, they have published almost 1,700 articles, including one in *Science* or *Nature* every month (on average) since the Institute's launch in 2009. In total, as of June 2017, we also have submitted close to 2,000 patent filings (with 120 patents issued), launched 19 new startup companies (e.g., *Emulate Inc., ReadCoor Inc., Ultivue Inc., Opsonix Inc., SLIPS Technologies Inc*.) and closed on nine additional licensing deals. The Institute with its 20 core and 16 associate faculty is currently responsible for producing a significant portion of all patent submissions and new startups across all operating units of Harvard University.

Beyond the metrics above, the Institute's impact lies in enabling the accelerated and successful translation of a number of key breakthroughs and technologies. One example is the invention of human Organs on Chips, which are microfluidic cell culture devices that were developed as replacements for animal testing. Following the first publication in *Science* in 2010 describing the human breathing lung‐on‐a‐chip,^10^ the technology's rapid development from demonstration of the first functional prototypes to multiple human Organs‐on‐Chips that can be integrated on a common automated instrument platform speaks to the Institute's ability to translate academic innovation into commercially valuable technologies in a big and meaningful way. By putting together a multidisciplinary team at the Institute comprised of faculty, students, fellows, Advanced Technology Team staff (with past industrial experience in this area) and an entrepreneur‐in‐residence, while leveraging grant support from the Defense Advanced Research Projects Agency (DARPA), Food and Drug Administration (FDA), and National Institutes of Health (NIH), the Wyss Institute team developed more than ten different Organs‐on‐Chip models, including chips that mimic lung, liver, gut, kidney, and bone marrow. In addition, the team engineered an instrument that automates chip operations and fluidically links the different organs‐on‐chips together to more closely mimic whole body physiology, while permitting high‐resolution imaging and molecular analysis. The Wyss Institute team also refined their technology and validated it for market need and impact by testing existing drugs and modeling various human diseases on‐chip in partnerships with major pharmaceutical companies. And with an eye toward creating a technology that can be mass‐manufactured cost effectively outside the lab, they formed additional industrial partnerships to achieve this goal and thereby, increased its likelihood of success in the marketplace. The transition of the Organs‐on‐Chips technology to the startup, Emulate Inc., was further enabled by the Wyss Institute's entrepreneur‐in‐residence and business development staff in collaboration with Harvard's OTD. The Institute's translation engine has—in a similar fashion‐enabled the translation of numerous technologies,^24^ including a method (FISSEQ) for simultaneously sequencing and mapping thousands of RNAs within cells and tissues to advance development of diagnostics and discover new drug targets^25^; a new approach to super‐resolution imaging by leveraging the Institute's DNA‐PAINT technology, which leverages transient binding of fluorophores to specific targets mediated by defined DNA–DNA interactions^16^; and a family of materials and coatings that repel virtually all fluids and biological fouling agents in industrial, medical, and consumer applications.^6^, ^26^

## 7 CONCLUSION

In less than 10 years, the Wyss Institute has created a culture and community within academia where risky ideas are encouraged, supported, and incubated, as well as where failures are accepted as much as successes. This allows for all intellectual knowledge to be kept in the organization and ultimately be turned into successful learning opportunities. The Institute's ability to catalyze true convergence of engineering, biology, medicine, and the physical sciences, while focusing on multiple high value applications in both medicine and nonmedical areas that can have broad impact on the world, is a unique differentiator relative to other Institutes and Centers that leverage a single scientific strength (e.g., genomics) and/or focus on a narrow research area (e.g., a particular disease, organ system, or a specific type of robot). But the real strength of the Wyss Institute model lies in the powerful network of its people, with an unprecedented ability to harness new ideas and intellectual property spawning from one high‐risk project, morphing onto multiple new projects, resulting in discovery of new high‐value problems and technology solutions that no one knew existed at the beginning. While there is no doubt that what we accomplished was made possible by the large scale of the philanthropic gift that launched our effort, we believe that there are many lessons we have learned that could translate to other academic centers around the world that want to similarly transform their academic inventions into new diagnostic and therapeutic products that will help patients, if the correct organizational and management structures are supported by institutional leadership.

Looking ahead, financial sustainability is a critical challenge for the Institute. While the translation of technologies to the marketplace is already resulting in increasing revenue from royalties, the Institute shares this revenue stream with Harvard University and its collaborating institutions in a win–win arrangement that aligns the interests of all consortium members. Thus, ensuring a steady and robust flow of additional funds from philanthropic and governmental sources remains a high priority. Having learned from the experience of the last eight and a half years, optimizing and further refining the Institute's model of translation is a key task ahead. Finally, ensuring that the dynamic and agile culture of the Institute stays strong as its technologies, people, and external environment evolve is an ongoing objective, which is critical for its long‐term success.

## DISCLOSURES

D.E.I. holds equity in *Emulate Inc., Opsonix Inc*., *FreeFlow Medical Devices Inc*., and *SlipChips Corp*., and is a member of their scientific advisory boards.
